# Nicotinamide protects against diabetic kidney disease through regulation of Sirt1

**DOI:** 10.1007/s12020-024-03721-7

**Published:** 2024-03-06

**Authors:** Yeping Yang, Jinya Huang, Lijie Xie, Yilin Wang, Shizhe Guo, Meng Wang, Xiaoqing Shao, Wenjuan Liu, Yi Wang, Qin Li, Xia Wu, Zhaoyun Zhang, Fangfang Zeng, Wei Gong

**Affiliations:** 1 Division of Endocrinology and Metabolism, Huashan Hospital, Fudan University, Shanghai, 200040 China; 2 Division of Endocrinology and Metabolism, Shanghai Ninth People’s Hospital, Shanghai JiaoTong University School of Medicine, Shanghai, 200011 China; 3 Department of Endocrinology and Metabolism, Jing’an District Center Hospital of Shanghai, Shanghai, 200040 China; 4 Institute of Endocrinology and Diabetology, Fudan University, Shanghai, 200040 China

**Keywords:** Nicotinamide, Diabetic kidney disease, Proximal tubule, Fibrosis, Oxidative stress, Sirtuin 1

## Abstract

**Purpose:**

To investigate the effect of nicotinamide (Nam) on diabetic kidney disease (DKD) in mice and explore its mechanism.

**Methods:**

Thirty DBA/2 J mice were randomly assigned to three groups. After 8 weeks of hyperglycemia induced by streptozocin (STZ), Nam and saline were administrated to STZ + Nam and STZ + NS mice, respectively, for 8 weeks. Non-diabetic mice (NDM) were used as control group. Twenty In2^−/−^ Akita mice were randomly divided into two groups. After 8 weeks of hyperglycemia, Nam and saline were administered to Akita + Nam and Akita + NS mice, respectively, for 6 weeks. Wild-type littermates were used as control group. Markers of renal injury were analyzed, and the molecular mechanisms were explored in human proximal tubular HK2 cells.

**Results:**

Urinary albumin-to-creatinine ratio (UACR) and kidney injury molecule 1 (KIM-1) decreased in the STZ + Nam and Akita + Nam groups. Pathological analysis showed that Nam improved the structure of glomerular basement membrane, ameliorated glomerular sclerosis, and decreased the accumulation of extracellular matrix and collagen. Compared to the diabetic control group, renal fibrosis, inflammation, and oxidative stress were reduced in the Nam-treated mice. The expression of sirtuin 1 (Sirt1) in human proximal tubular HK2 cells was inhibited by high glucose and Nam treatment enhanced its expression. However, in HK2 cells with Sirt1 knockdown, the protective effect of Nam was abolished, indicating that the beneficial effect of Nam was partially dependent on Sirt1.

**Conclusions:**

Nam has a renoprotective effect against renal injury caused by hyperglycemia and may be a potential target for the treatment of DKD.

## Introduction

Diabetic kidney disease (DKD), a major cause of morbidity, mortality, and economic hardship globally, is on the rise [1]. Despite current therapies, there is a high risk of DKD onset and progression. Therefore, widespread innovative treatments are urgently required to improve the health outcomes of patients with DKD.

Yasuda et al. demonstrated that the downregulation of sirtuin proteins, key molecules in energy metabolism, play essential roles in the progression of DKD concomitantly with the dysregulation of nicotinamide adenine dinucleotide (NAD^+^) metabolism in the proximal tubule [2]. Sirtuin 1 (Sirt1) is one of the NAD^+^-dependent protein deacetylases that has been shown to be involved in protection against various kidney injuries including DKD [3, 4]. Sirt1 can inhibit NF-κB- and STAT3-induced inflammatory responses [5] and suppress p53-related apoptosis in diabetic kidneys [6]. Sirt1 reactivation is expected to provide a protective effect against DKD. NAD^+^ is an important molecule in cellular respiration and ATP-generating reactions, and functions as a coenzyme for oxidoreductases and dehydrogenases. NAD^+^ deficiency is one of the initiating factors in aging and metabolically induced pathological changes, including diabetes [7, 8]. Therefore, increasing NAD^+^ concentrations may improve DKD.

In terms of supplementation with NAD^+^ precursors, nicotinamide mononucleotide (NMN) and nicotinamide riboside (NR) have fewer adverse reactions, and can efficiently enhance NAD^+^ biosynthesis [9] to improve various metabolic abnormalities. In the type 2 diabetic db/db model, NMN administration at doses of 300 mg/kg and 500 mg/kg improved albuminuria, whereas 100 mg/kg NMN had no effect [10]. However, some potential side effects of NMN have been proposed, particularly with high-dose administration, such as hepatic pressure and cancer growth [11]. NR has been reported to prevent and alleviate glucose intolerance in insulin resistant models [12, 13]. However, recent clinical studies have shown that NR supplementation fails to improve glucose tolerance and pancreatic function markers in obese, pre-diabetic individuals. It is important to note that NR is largely degraded in the gastrointestinal tract [14] and circulation [15] to form nicotinamide (Nam) or nicotinic acid (NA).

Nam is an amino compound of vitamin B3 and is an important precursor of NAD^+^. Nam can be synthesized by the body or ingested through intake of meat, fish, beans, nuts, coffee, tea, etc. Nam is a specific regulator of mouse islet progenitor cell (IPC) differentiation, which produces cells that exhibited many characteristics of adult β cells, including the ability to ameliorate preclinical diabetes and possessing a highly comparable transcriptome profile [16]. Nam also regulates human IPC differentiation, improves renal fibrosis in rat unilateral ureteral obstruction model [17, 18], prevents acute kidney injury [19], and inhibits renal inflammation in mice with systemic lupus erythematosus [20]. However, no relevant studies have been conducted on Nam and DKD, and the efficacy of Nam in DKD remains unclear.

In the current study, we showed that exogenous supplementation with Nam could delay kidney disease progression in diabetic mice, protect the renal structure, reduce glomerular and tubular injury, and ameliorate renal fibrosis and oxidative stress. Moreover, these effects are dependent on Sirt1.

## Materials and methods

### Animal models

All animal studies were performed in accordance with protocols approved by the Institutional Animal Care and Use Committee at Fudan University. 5-week-old male DBA/2 J mice (obtained from Shanghai Laboratory Animal Center, Shanghai, China) were injected with streptozocin (STZ, 50 mg/kg, i.p.) on 5 consecutive days. After 8 weeks of diabetes, which defined as blood glucose (BG) > 16.7 mmol/L, the mice were randomly separated into either a Nam-treated group (*n* = 10, intraperitoneally injected with Nam 400 mg/kg/d, STZ + Nam group) or a control group treated with saline (*n* = 10, 10 mL/kg/d, STZ + NS group). After 8 weeks of treatment, the mice were sacrificed. Another group of normal DBA/2 J mice (*n* = 10, NDM group) was administered saline (10 mL/kg/d) and used as a control. To generate another type 1 diabetic mouse model, twenty 5-week-old In2^-/-^ Akita mice (obtained from the Model Animal Research Center, MARC, Nanjing University, Nanjing, China) were randomly divided into two groups: mice intraperitoneally injected with 400 mg/kg/d Nam (Akita + Nam) or 10 mL/kg/d saline (Akita + NS). Littermate wild-type C57BL/6 male mice (*n* = 10, WT group) were administered saline (10 mL/kg/d) as a control. After 8 weeks of hyperglycemia, Nam and saline were administrated to Akita + Nam and Akita + NS mice, respectively, for 6 weeks. Mice were housed in a temperature-controlled room and provided water and standard laboratory chow ad libitum during the entire study period.

Body weight (BW) and BG were measured weekly, and kidney weight (KW) was measured when the mice were sacrificed. Blood pressure (BP) was measured monthly using tail-cuff plethysmography (BP-2000; Softron Beijing Incorporated, Beijing, China). Prior to sacrifice, the mice were housed in metabolic cages, and 24-h urine samples were collected. Urinary albumin levels were measured using an enzyme-linked immunosorbent assay (Bethyl Laboratory Inc., Houston, TX, USA). Urinary creatinine concentration was determined using a chemiluminescence assay (Exocell, Philadelphia, PA, USA), and the urinary albumin-to-creatinine ratio (UACR) was calculated. Blood was collected from the left heart chamber and centrifuged at 3000 g for 10 min at 4 °C for serum isolation. Serum total cholesterol, serum triglycerides, kidney injury molecule 1 (KIM-1), and neutrophil gelatinase-associated lipocalin (NGAL) levels were measured by enzyme-linked immunosorbent assay (Meimian Biotechnology, Yancheng, China). The serum creatinine concentration was measured using a chemiluminescence assay (Exocell, Philadelphia, PA, USA).

### Cell culture and transfection

Human proximal tubular HK2 cells were cultured in DMEM supplemented with 10% fetal bovine serum (FBS) at 37 °C in 5% CO_2_. HK2 cells were exposed to DMEM containing 5.6 mM (normal glucose, NG) or 30 mM (high glucose, HG) glucose. A concentration of 10 nM was used for Nam supplementation according to a previous study [21]. Sirt1 was knocked down by transfection of small interfering RNA (siRNA) using Lipofectamine 3000 (Invitrogen, Life Technologies, Carlsbad, USA) according to the manufacturer’s instructions. Sirt1 and scramble siRNAs were purchased from GenePharma Corporation (Shanghai, China). The sequences of the siRNAs are: Sirt1 siRNA, 5′-GAAGUUGACCUCCUCAUUG-3′ and 3′-CAAUGAGGAGGUCAACUUC-5′; and scramble siRNA, 5′-UUCUCCGAACGUGUCACGUTT-3′ and 3′-ACGUGACACGUUCGGAGAATT–5′.

### Histology

Partial cortices of the kidneys were fixed overnight with 4% paraformaldehyde. The tissues were embedded in paraffin, stained with hematoxylin and eosin (H&E), periodic acid–Schiff (PAS), or Masson’s trichrome reagent, and examined under an optical microscope. Glomerular sclerosis index was calculated as previously described [22]. Renal tissue was examined using transmission electron microscopy (TEM) after fixation with 10% glutaraldehyde. The glomerular basement membrane (GBM) thickness in the TEM images was measured using ImageJ software. The method used to measure the width of the foot processes has been previously described [22]. Foot-process effacement was determined as the length of the effacement divided by the length of the capillary. For three mice in each experimental group, five glomeruli per mouse and five random open capillary loops per glomeruli were measured using ImageJ software. For the quantification of endothelial fenestrations, values were expressed as the mean number of endothelial fenestrations per 100 μm of the capillary loop perimeter.

### Immunohistochemistry (IHC)

The expression of TGF-β1 (ab92486, Abcam, Cambridge, UK), E-cadherin (20874-1-AP, Proteintech, Rosemont, USA), α-smooth muscle actin (α-SMA; 55135-1-AP, Proteintech), collagen I (Col I; ab34710, Abcam), and nitrotyrosine (sc32757, Santa Cruz, Dallas, TX, USA) was detected using an established method [23]. Ten fields for each section were imaged. The ratio of integrated optical density to area was quantified using ImageJ software.

### RNA extraction and quantitative polymerase chain reaction (qPCR)

The total RNA of the renal cortex was isolated, reverse transcribed to cDNA, and used to measure the expression of E-cadherin, α-SMA, TGF-β1, Collagen I (Col I), nuclear factor erythroid 2-related factor 2 (Nrf2), catalase (CAT), superoxide dismutase 1 (SOD1), SOD2, glutathione peroxidase (GPx), Sirt1, Sirt3, Sirt6, Collagen IV (Col IV) and β-actin using qPCR [24]. The sequences of the primers used in this study are listed in Supplementary Table 1. Each sample was run in triplicate and normalized to β-actin levels.

### Protein extraction and Western blot analysis

Protein expression in the renal cortex or in HK2 cells was determined using a published protocol [25]. The primary antibodies used were against TGF-β1 (ab92486, Abcam), E-cadherin (20874-1-AP, Proteintech), α-SMA (55135-1-AP, Proteintech), Col I (ab34710, Abcam), nitrotyrosine (sc32757, Santa Cruz), Sirt1 (ab110314, Abcam), heme oxygenase 1 (HO-1, sc-390991, Santa Cruz), Col IV (ab6586, Abcam) and β-actin (23660-1-AP, Proteintech).

### Detection of intracellular reactive oxygen species (ROS)

HK2 cells were cultured in 96-well chamber slides in growth medium. After incubating in the pre-warmed buffer containing 10 μM DHE-probe (Vigorous Biotechnology, Beijing, China) at 37 °C for 30 min, the buffer was removed and the fluorescence intensity was determined with an excitation wavelength of 480 nm and an emission wavelength of 600 nm.

### Statistical analysis

All data are expressed as mean ± SEM from three independent experiments. Statistical analysis was performed using the statistical package SPSS for Mac Version 20.0 (SPSS, Inc., Chicago, IL, USA). The significance of the differences among the different groups was evaluated using one-way analysis of variance (ANOVA) followed by Tukey’s test. Statistical significance was set at *P* < 0.05.

## Results

### Animal characteristics

Data on BW, kidney weight to body weight (KW/BW), BG, serum total cholesterol (TCH), serum triglyceride (TG), and BP in STZ-induced mice are summarized in Table 1. The weekly BW and BG records of the mice are shown in Supplementary Fig. 3. The BW levels in the STZ + NS and STZ + Nam groups were significantly lower than those in the NDM group. The BG, TCH, and TG levels were significantly higher in the STZ + NS and STZ + Nam groups than in the NDM group. However, there was no difference between the STZ + NS and STZ + Nam groups. Compared with the NDM group, the KW/BW ratio increased in the STZ + NS group, which was reversed in the STZ + Nam group. Systolic and diastolic BPs showed no statistically significant differences among the three groups. The basic characteristics of Akita mice are shown in Supplementary Table 2. Nam had no impact on BW, BG, BP, TCH, or TG in Akita mice.

**Table 1:** Basic characteristics of STZ-induced diabetic mice

	NDM (*n* = 10)	STZ + NS (*n* = 9)	STZ + Nam (*n* = 8)	*F* value	*P* value
BW (g)	27.48 ± 0.19	20.36 ± 0.13^***^	21.01 ± 0.25^***^	43.73	<0.001
KW/BW (mg/g)	23.24 ± 0.26	28.83 ± 0.47^***^	27.58 ± 0.24^*^	9.36	<0.001
BG (mM)	6.02 ± 0.09	29.37 ± 0.30^***^	30.21 ± 0.49^***^	219.00	<0.001
TCH (mM)	2.02 ± 0.12	4.42 ± 0.48**	4.64 ± 0.32**	9.63	0.0013
TG (mM)	0.98 ± 0.08	2.97 ± 0.12**	3.03 ± 0.13**	11.67	<0.001
SBP (mmHg)	110.48 ± 1.56	106.89 ± 2.14	102.01 ± 2.06^**^	4.90	0.008
DBP (mmHg)	56.36 ± 1.97	56.43 ± 2.10	51.88 ± 1.63	1.78	0.17

*BW* blood weight, *BG* blood glucose, *TCH* total cholesterol, *TG* triglyceride, *KW* kidney weight, *SBP* systolic blood pressure, *DBP* diastolic blood pressure

Data are mean ± SEM. ***means *P* < 0.001; **means *P* < 0.01; compared to NDM group

### Nam reduced urinary albumin and proximal tubular injury in diabetic kidney

**Fig. 1:**
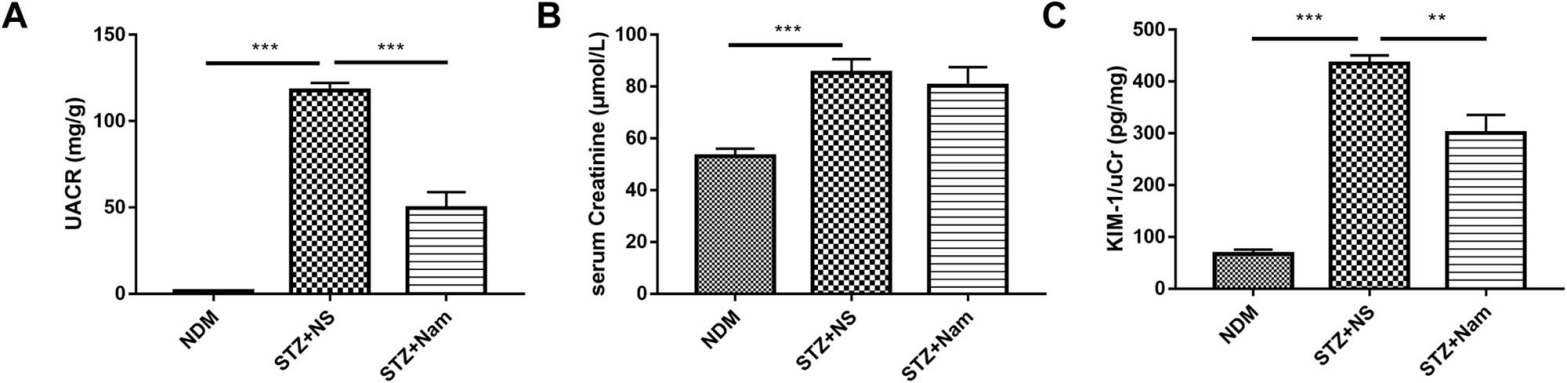
Nam reduces urinary albumin and proximal tubular injury in diabetic kidney. **A** The UACR, **B** serum creatinine levels and **C** urinary KIM-1/uCr in STZ-induced diabetic mice. **p < 0.01; ***p < 0.001; UACR urinary albumin-to-creatinine ratio, uCr urinary creatinine, NDM nondiabetic control, STZ + NS diabetic control, STZ + Nam diabetic mice treated with Nam

Compared with the NDM group, the STZ + NS group showed significantly increased UACR (1.66 ± 0.23 mg/g vs 117.68 ± 4.46 mg/g, *P* < 0.0001), which was reduced by Nam administration (117.68 ± 4.46 mg/g vs 49.85 ± 9.09 mg/g, *P* < 0.0001) (Fig. 1A). Serum creatinine (SCr) markedly increased in the STZ + NS (85.28 ± 5.32 vs. 53.17 ± 2.88, *P* < 0.0001) and STZ + Nam (80.39 ± 7.17 vs. 53.17 ± 2.88, *P* = 0.0015) groups when compared with the NDM group (Fig. 1B). Mice in the STZ + NS group had significantly higher urinary KIM-1/urinary creatinine (uCr) levels than those in the NDM group, whereas Nam significantly decreased KIM/uCr levels (Fig. 1C). A similar effect was observed in Akita mice (Supplementary Fig. 1).

### Nam protected the kidney structure in DKD

**Fig. 2:**
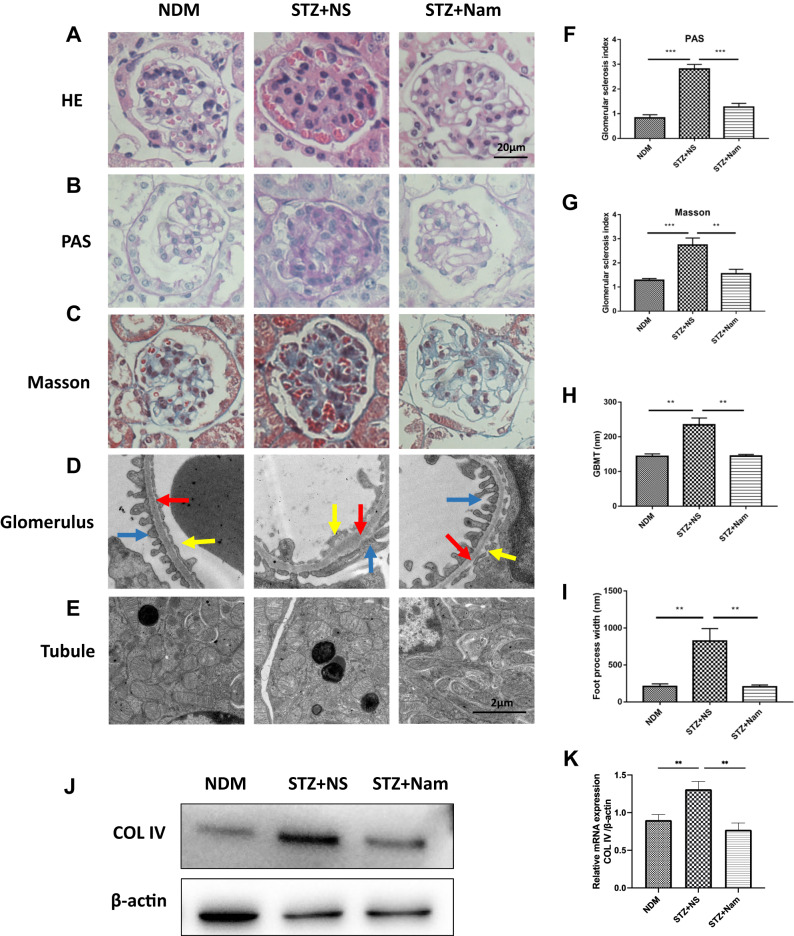
Nam protected the kidney structure in DKD. Representative images of renal tissue stained with **A** HE, **B** PAS and **C** Masson in all mice. **D** Representative electronic micrographs of the GBM and fenestrae of endothelial cells. Red arrow: basement membrane, yellow arrow: fenestrae of endothelial cells, blue arrow: foot process of podocyte. **E** Representative electronic micrographs of the tubules. **F, G** The glomerular sclerosis indexes for PAS and Masson staining, respectively. **H, I** The quantitative thickness of the GBM and foot process width for these 3 groups. **p < 0.01; ***p < 0.001; **J** Protein expression of Col IV for 3 groups of mice. **K** Gene expression of Col IV for 3 groups of mice; NDM nondiabetic control, STZ + NS diabetic control, STZ + Nam diabetic mice treated with Nam

H&E, PAS, and Masson’s trichrome staining were used to examine pathological changes in the kidney tissues (Fig. 2A–C). H&E staining showed glomerular hypertrophy, cellular and stromal hyperplasia in the mesangial in STZ + NS mice compared to NDM mice, whereas exogenous Nam reversed these pathological changes (Fig. 2A). The percentages of PAS- or Masson’s trichrome-positive area, indicative of mesangial expansion and collagen deposition, respectively, and the glomerular sclerosis index were significantly increased in the diabetic glomeruli compared with those of the NDM controls (Fig. 2F, G). However, these phenotypes were significantly attenuated in the diabetic mice treated with Nam. Similar results for PAS and Masson’s trichrome staining were observed in the Akita mice (Supplementary Fig. 2).

Previous studies have demonstrated that an increase in GBM thickness is one of the most important pathological changes associated with DKD [26, 27]. Glomerular basement membrane is mainly constituted of Collagen IV (COL IV), we found that the mRNA and protein expression of COL IV were upregulated in STZ + NS group, which were restored by 8-weeks of Nam treatment (Fig. 2J and K). Moreover, we examined the glomerular ultramicrostructure using TEM to verify whether Nam could protect the glomeruli of diabetic mice. Our results showed that GBM thickness was significantly increased in the STZ + NS group compared with that in NDM mice, whereas Nam protected against this pathologic change (Fig. 2D, red arrow). This was confirmed by a semi-quantitative analysis (Fig. 2H). Nam also alleviated the fusion of podocyte foot processes (Fig. 2D, blue arrow), which partially explains the protective role of Nam against albuminuria. Similar results were observed in the Akita mice (Supplementary Fig. 2).

Recent studies confirmed that renal tubular injury also plays an important role in diabetic renal injury. To verify whether Nam could protect the tubules of diabetic mice, we examined the tubular ultramicrostructure using TEM. The mitochondria in the renal tubules of NDM mice were fusiform or round, with tightly arranged mitochondrial ridges and a complete mitochondrial envelope. Mitochondrial swelling, mitochondrial ridge loosening, and mitochondrial envelope damage in the renal tubules were observed in the STZ + NS group. After Nam intervention, the morphological and structural abnormalities of the mitochondria were improved (Fig. 2E). Similar results were observed in the Akita mice (Supplementary Fig. 2).

### Nam reduced renal tubular epithelial transdifferentiation and fibrosis in diabetic mice

**Fig. 3:**
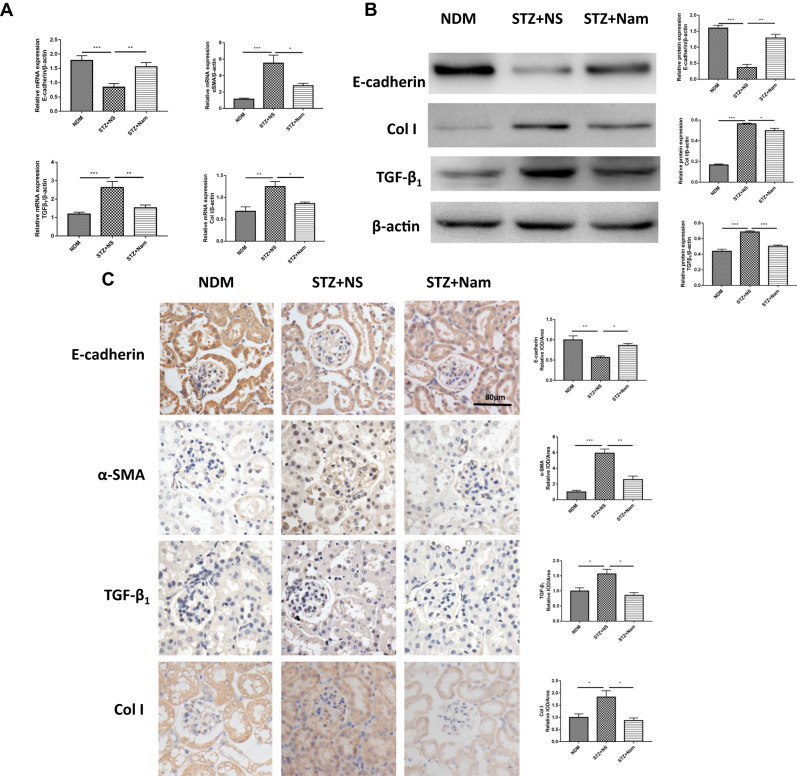
Nam reduces renal tubular epithelial transdifferentiation and fibrosis in diabetic mice. **A** Gene expression of E-cadherin, α-SMA, TGF-β1 and Col I for 3 groups of mice. **B** Protein expression of E-cadherin, TGF-β1 and Col I for 3 groups of mice. **C** IHC staining of E-cadherin, α-SMA, TGF-β1 and Col I for 3 groups of mice. *p < 0.05; **p < 0.01; ***p < 0.001; NDM nondiabetic control, STZ + NS diabetic control, STZ + Nam diabetic mice treated with Nam

E-cadherin is a connective protein between renal tubular epithelial cells. α-SMA is a specific marker of mesenchymal fibroblasts. These two markers were used here to reflect the renal tubular epithelial transdifferentiation. We found that E-cadherin gene expression was lower in STZ + NS mice than in NDM mice. However, it was increased in STZ + Nam mice compared to STZ + NS mice. In contrast, the gene expression of α-SMA was increased in STZ + NS mice and decreased in STZ + Nam mice compared with that in NDM mice (Fig. 3A).

Western blot analysis (Fig. 3B) showed similar results. E-cadherin protein expression levels in the STZ + NS group were significantly lower than those in the NDM group, which were restored with Nam treatment. IHC showed that renal expression of α-SMA was very low in NDM mice, whereas E-cadherin was widely expressed in the cytoplasm of renal tubules (Fig. 3C). In STZ + NS mice, the renal expression of α-SMA was mainly concentrated in the cytoplasm of renal tubules and was significantly increased compared to that in NDM mice, whereas the expression of E-cadherin was significantly decreased. In the STZ + Nam group, α-SMA expression level was significantly lower than that in the STZ + NS group, whereas the E-cadherin expression was elevated compared with STZ + NS group.

To verify whether Nam improved renal fibrosis, we examined renal TGF-β1 and Col I with qPCR, Western blot and IHC. Compared with the NDM group, STZ + NS mice showed significantly increased gene expressions of TGF-β1 and Col I, which were reduced in the STZ + Nam mice (Fig. 3A). We also found that the protein expressions of TGF-β1 and Col I were decreased by Nam treatment (Fig. 3B). Similar IHC results of TGF-β1 and Col I in STZ-induced mice are shown in Fig. 3C.

### Nam reduced renal oxidative stress in diabetic mice

**Fig. 4:**
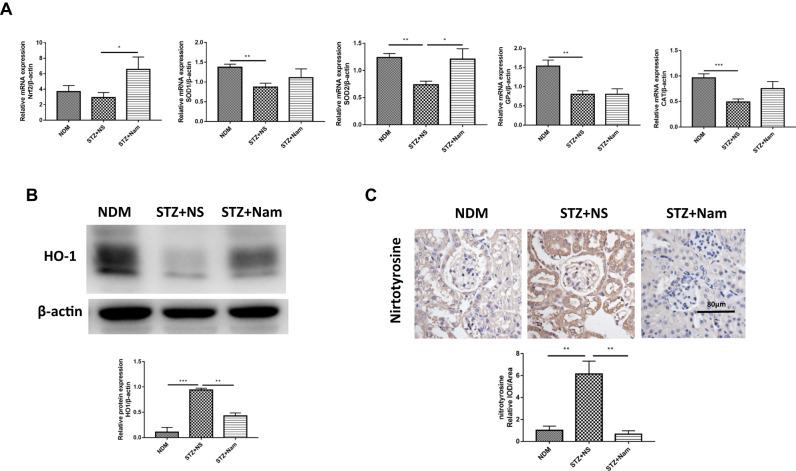
Nam reduced renal oxidative stress in diabetic mice. **A** Gene expression of Nrf2, SOD1, SOD2, GPx and Cat for 3 groups of mice. **B** Protein expression of HO-1 for 3 groups of mice. **C** IHC staining of Nitrotyrosine for 3 groups of mice. *p < 0.05; **p < 0.01; ***p < 0.001; NDM nondiabetic control, STZ + NS diabetic control, STZ + Nam diabetic mice treated with Nam

Nrf2 is a key transcription factor that regulates the expression of various antioxidant proteins in vivo and plays an important role in tissue damage induced by oxidative stress. qPCR results showed that, compared with the STZ + NS group, the expression of Nrf2 mRNA significantly increased after Nam intervention (Fig. 4A).

The expression of the antioxidant enzymes CAT, GPx, SOD1, SOD2, and HO-1 were measured to assess oxidative stress. Compared to NDM mice, the renal gene expression levels of CAT, GPx, SOD1, and SOD2 in STZ + NS mice was significantly decreased, but the SOD2 expression was significantly increased in STZ + Nam mice (Fig. 4A). Western blotting showed that the renal expression of HO-1 in STZ + NS mice was significantly lower than that in NDM mice, and the expression of HO-1 in STZ + Nam mice was significantly higher than that in NDM mice (Fig. 4B).

Nitrotyrosine is the product of oxidation of tyrosine groups in proteins. Elevated nitrotyrosine levels are characteristic of excessive accumulation of oxidized products in vivo. Renal IHC staining showed that nitrotyrosine accumulated in glomerular sacs and tubular epithelial cells in STZ + NS mice, whereas in STZ + Nam mice, nitrotyrosine content was significantly reduced (Fig. 4C).

### Nam attenuated high glucose-induced oxidative stress and fibrosis via Sirt 1

**Fig. 5:**
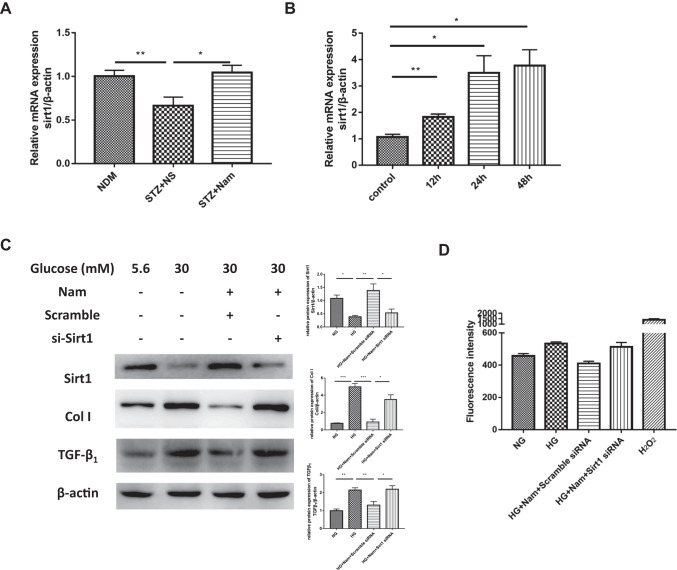
Nam attenuated high glucose-induced oxidative stress and fibrosis via Sirt1. **A** Gene expression of Sirt1 for 3 groups of mice. **B** Gene expression of Sirt1 in HK2 cells treated with Nam. **C** Protein expression of Sirt1, Col I and TGF-β1 in HK2 cells. **D** Detection of intracellular ROS in HK2. *p < 0.05; **p < 0.01; ***p < 0.001; NDM, nondiabetic control; STZ + NS, diabetic control, STZ + Nam, diabetic mice treated with Nam

To explore the potential mechanism by which Nam protects against DKD, we focused on the sirtuins family, which have broad enzymatic activities that cover multiple cellular functions. Herein, we examined the expression of Sirt1, Sirt3 and Sirt6, which are abundantly expressed in the kidney. Our findings demonstrated that Sirt3 and Sirt6 were downregulated in the kidneys of diabetic mice, but Nam treatment did not show significant impact on the mRNA level of Sirt3 and Sirt6 (Supplementary Fig. 4A). So we speculated that the beneficial effect of Nam depends, at least in part, on Sirt1, which is regulated downstream of Nam. It is interesting that the mRNA and protein levels of Sirt1 were downregulated in diabetic kidneys compared to the NDM group, whereas Nam significantly upregulated Sirt1 expression in the kidneys of diabetic mice (Fig. 5A). In vitro experiments also showed that the mRNA level of Sirt1 was upregulated in a time-dependent manner in HK2 cells (Fig. 5B).

A potential relationship between Nam and Sirt1 was detected in vitro. As shown in Fig. 5C, compared to cells cultured with a normal glucose concentration (5.6 mM), those cultured in a high glucose concentration (30 mM) had lower Sirt1 levels while TGF-β1 and Col I levels were upregulated. After the administration of Nam, the levels of TGF-β1 and Col I returned to levels comparable to cells cultured with normal glucose levels (5.6 mM) (Fig. 5C). When Sirt1 was knocked down, the levels of TGF-β1 and Col I were no longer affected by Nam treatment (Fig. 5C). Similarly, ROS accumulation in HK2 cells exposed to high glucose was improved by supplementation with Nam, but knockdown of Sirt1 eliminated the effectiveness of Nam (Fig. 5D). In summary, Sirt1 mediated the nephroprotective effect of Nam under high glucose conditions.

## Discussion

To the best of our knowledge, this is the first study to investigate the protective effect of Nam on DKD in type 1 diabetic mice using two different mouse models: STZ-induced diabetic mice and Akita mice. The results showed that Nam significantly protected glomerular and tubular function, maintained nephron ultrastructure, and improved pathological changes, such as oxidative stress and fibrosis. In addition, the in vitro studies showed that the protective effect of Nam in high glucose-induced HK2 cell injury is dependent on Sirt1.

DKD is characterized by persistent albuminuria and/or a decline in the glomerular filtration rate with clinical manifestations[28, 29]. Previous studies have reported that Nam can reduce urinary albumin excretion in preeclampsia mice [20], and preserve renal function in mice with acute kidney injury [30]. In this study, we demonstrated that Nam significantly reduced UACR, clarifying its benefits on the glomeruli in DKD. As a key part of the nephron, proximal tubule dysfunction is considered the driving force of DKD [31] and soluble KIM-1 in urine can be used as a biomarker of renal proximal tubular injury [32]. In this study, Nam significantly reduced the levels of KIM-1 in urine, indicating its protective effect on renal tubules. The ultrastructural lesions of DKD include GBM thickening, endothelial fenestration, effacement of glomerular podocytes foot processes, tubular atrophy, mitochondrial swelling, and tubulointerstitial fibrosis [33, 34]. To verify the protective effect of Nam on the diabetic kidney, structural changes were observed by TEM and light microscopy, which revealed preservation of the nephron structure. In summary, Nam effectively protected the function and structure of the glomeruli and tubules.

Oxidative stress plays a pivotal role in the development of DKD [35]. As an important transcription factor, Nrf2 protects against oxidative stress by upregulating endogenous antioxidants, including HO-1, SODs, and glutathione transferases [36–39]. We found that Nam upregulated the levels of Nrf2 and SOD2, eliminated nitrotyrosine in diabetic kidneys, and reduced the accumulation of ROS in HK2 cells under high-glucose conditions, which confirmed its antioxidant effect. In addition, our study confirmed that the role of Nam is dependent on Sirt1. Downstream of Nam, Sirt1 has a positive impact on mitochondrial function and oxidative stress responses [40] and can upregulate reductases such as CAT and SOD2 to clear ROS [41, 42]. Therefore, the use of Nam to raise the level of Sirt1 could delay the progression of DKD.

Renal fibrosis is related to activation and overexpression of TGF-β1 and is another important feature of DKD [43, 44]. Renal fibrosis is characterized by excessive collagen deposition in the extracellular matrix and accumulation of myofibroblasts in the kidney [45, 46]. During fibrosis, epithelial cells lose their characteristics, whereas mesenchymal cells accumulate in the kidney [47]. In our experiment, Nam inhibited the loss of E-cadherin and expression of α-SMA in the kidneys of STZ-induced diabetic mice, indicating that it can inhibit fibrosis. Previous studies have reported that Sirt1 can inhibit renal fibrosis induced by TGF-β1 signal transduction by deacetylating Smad3 and Smad4 [48, 49]. We found that Nam supplementation improved the suppressed Sirt1 levels in both diabetic kidney and high glucose-cultured HK2 cells, supporting the protective role of Nam in DKD.

Although there have been surprising discoveries about the role of Nam in DKD, the current findings have some limitations. First, the rodent model cannot completely simulate the pathological processes of DKD in humans. Second, the mechanism through which Nam regulates Sirt1 expression requires further investigation. Finally, since nephrons consist of several cell types, the effects of Nam on other renal cells need to be assessed.

## Conclusion

This study demonstrated that Nam can provide renal protection to diabetic mice, which may be attributed to the upregulation of Sirt1. These findings provide new insights for the treatment of DKD.

### Supplementary information

Supplementary figure

Supplementary Tables

Supplementary figures
