# Supplementary material for: Nicotinamide protects against diabetic kidney disease through regulation of Sirt1
Source: Endocrine. 2024 Mar 6;85(2):638–48. doi: 10.1007/s12020-024-03721-7 (PMC11291543; doi:10.1007/s12020-024-03721-7)
Supplement: Supplementary file 1 — Supplementary figure [file 12020_2024_3721_MOESM1_ESM.pptx]

## Slide 1
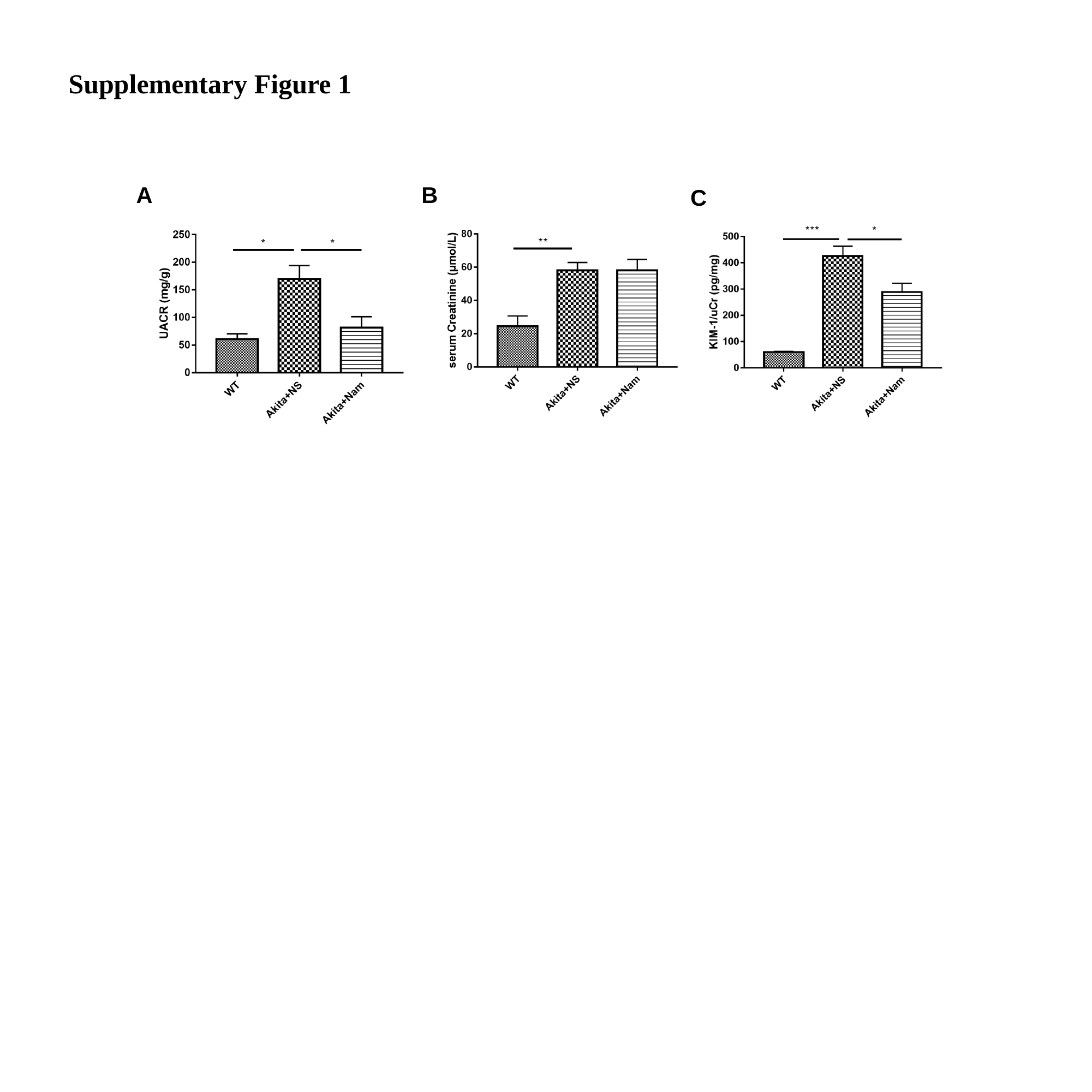

Supplementary Figure 1
A
B
C

## Slide 2
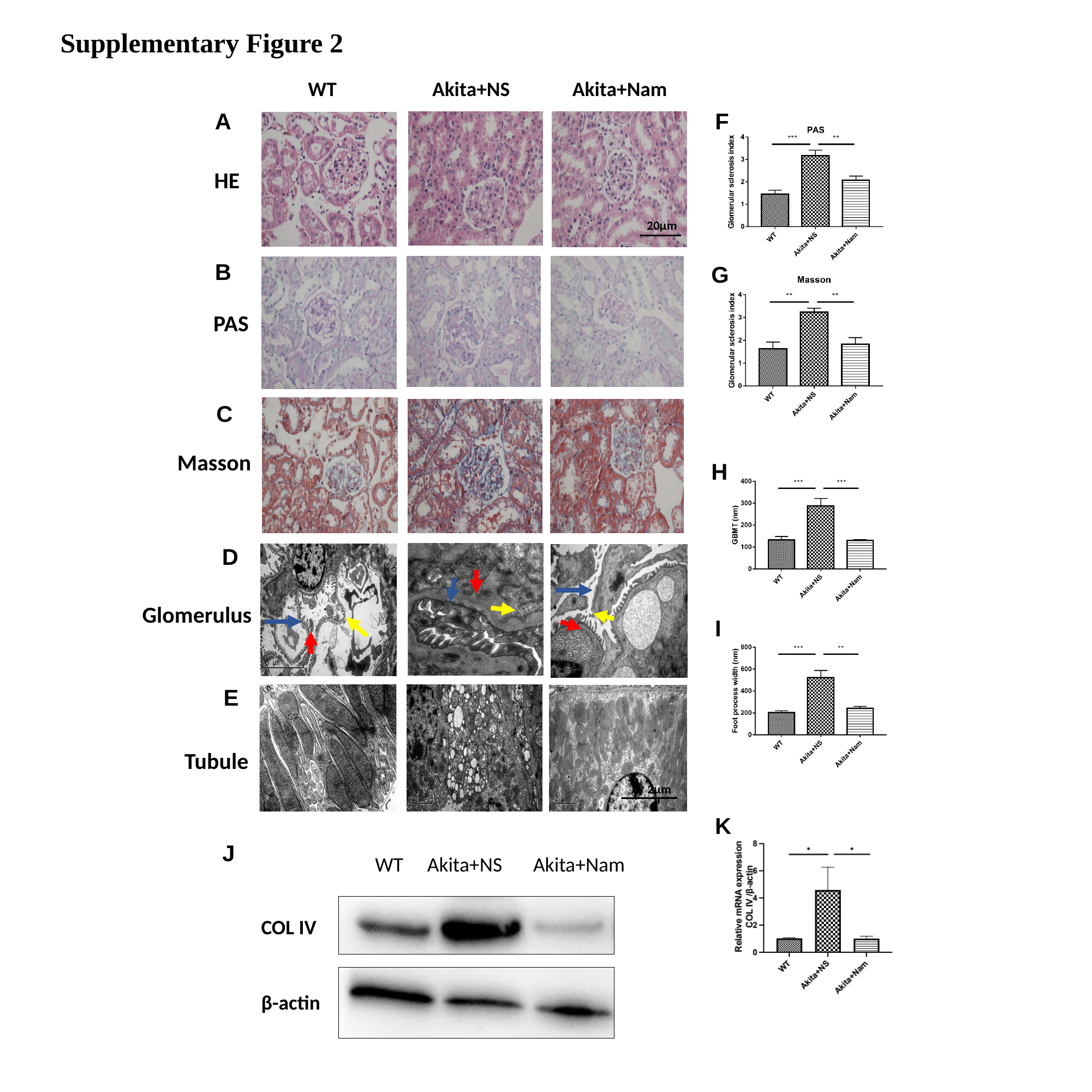

Supplementary Figure 2
| WT | Akita+NS | Akita+Nam |
| --- | --- | --- |
A
F
HE
20μm
B
G
PAS
C
Masson
H
D
Glomerulus
I
E
Tubule
2μm
K
J
| WT | Akita+NS | Akita+Nam |
| --- | --- | --- |
COL IV
β-actin

## Slide 3
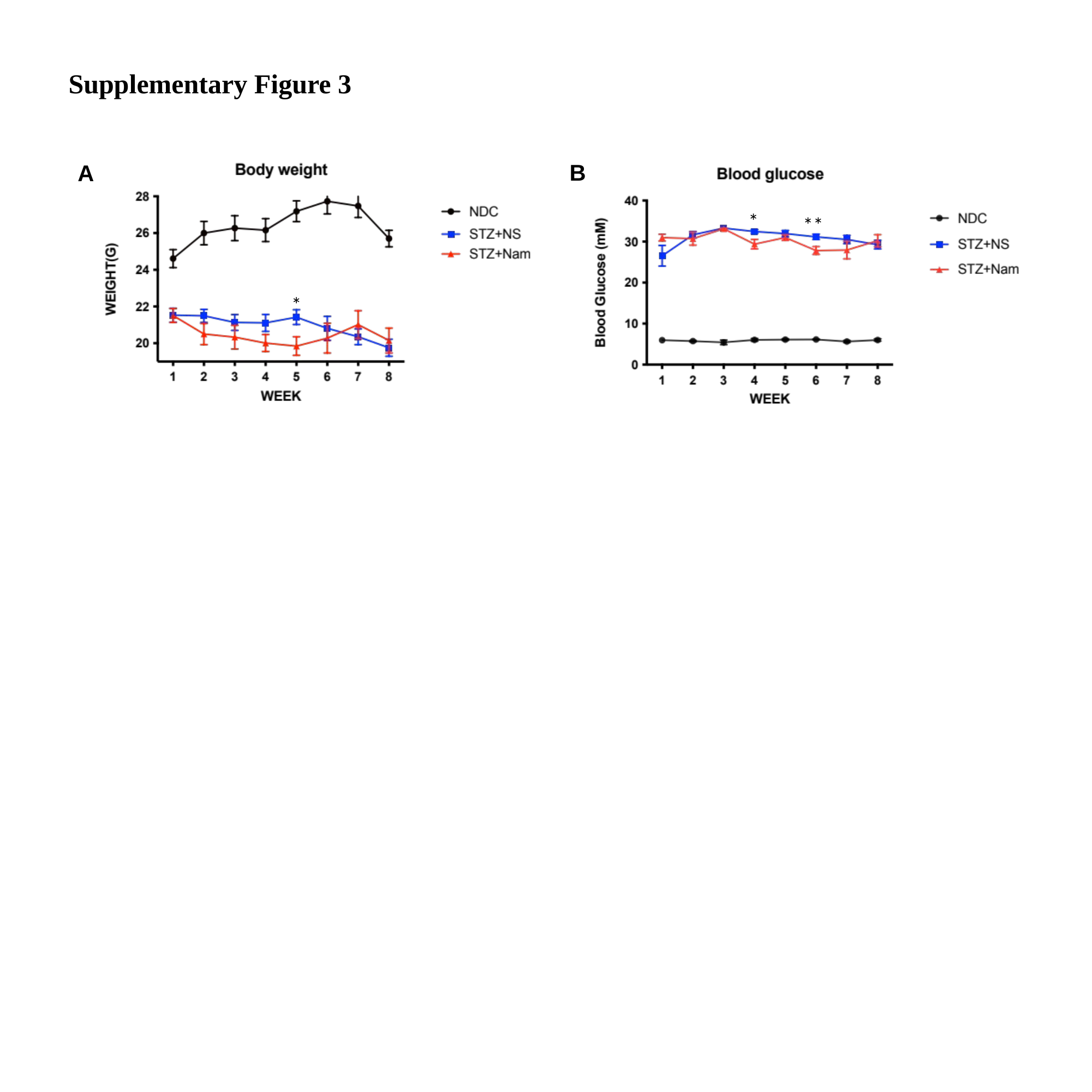

Supplementary Figure 3
B
*
**
A
*

## Slide 4
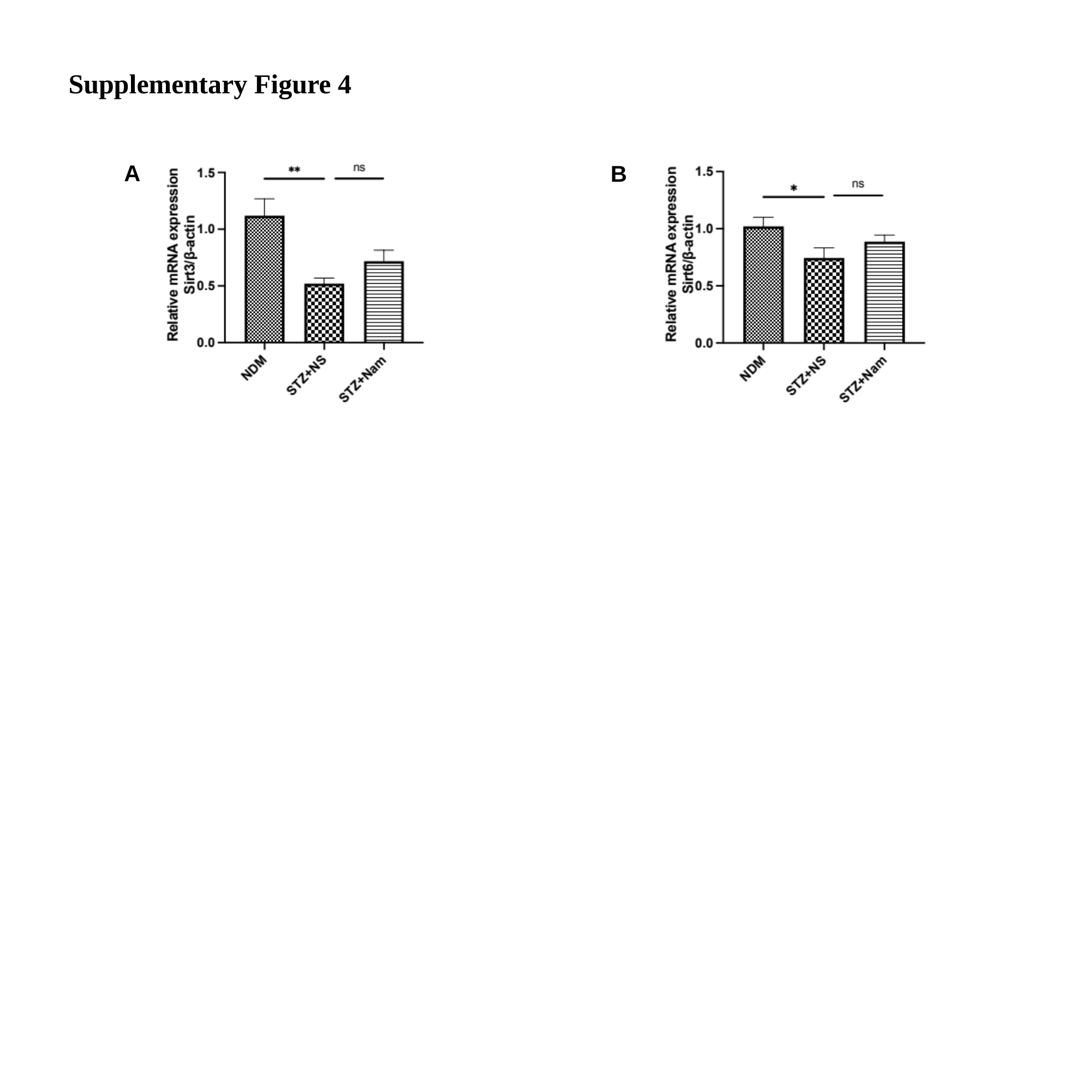

Supplementary Figure 4
A
B
