# Supplementary material for: Nicotinamide protects against diabetic kidney disease through regulation of Sirt1
Source: Endocrine. 2024 Mar 6;85(2):638–48. doi: 10.1007/s12020-024-03721-7 (PMC11291543; doi:10.1007/s12020-024-03721-7)
Supplement: Supplementary file 2 — Supplementary Tables [file 12020_2024_3721_MOESM2_ESM.docx]

Supplementary Table 1. Primers

| **Gene** | **Forward** | **Reverse** |
| --- | --- | --- |
| β-actin | GGCTGTATTCCCCTCCATCG | CCAGTTGGTAACAATGCCATGT |
| E-cadherin | CAGTTCCGAGGTCTACACCTT | TGAATCGGGAGTCTTCCGAAAA |
| ɑ-SMA | CCCAGACATCAGGGAGTAATGG | TCTATCGGATACTTCAGCGTCA |
| Col I | GCTCCTCTTAGGGGCCACT | ATTGGGGACCCTTAGGCCAT |
| TGF-β_1_ | CCACCTGCAAGACCATCGAC | CTGGCGAGCCTTAGTTTGGAC |
| Nrf2 | TAGATGACCATGAGTCGCTTGC | GCCAAACTTGCTCCATGTCC |
| Catalase | GGAGGCGGGAACCCAATAG | GTGTGCCATCTCGTCAGTGAA |
| SOD1 | AACCAGTTGTGTTGTCAGGAC | CCACCATGTTTCTTAGAGTGAGG |
| SOD2 | CAGACCTGCCTTACGACTATGG | CTCGGTGGCGTTGAGATTGTT |
| GPx | CCACCGTGTATGCCTTCTCC | AGAGAGACGCGACATTCTCAAT |
| Sirt1 | ATGACGCTGTGGCAGATTGTT | CCGCAAGGCGAGCATAGAT |
| Sirt3 | ATCCCGGACTTCAGATCCCC | CAACATGAAAAAGGGCTTGGG |
| Sirt6 | ATGTCGGTGAATTATGCAGCA | GCTGGAGGACTGCCACATTA |
| COL IV | CAAAGGCATCAGGGGAATAACT | ACCCTTAGATCCGTTGCATCC |
| β-actin* | CATGTACGTTGCTATCCAGGC | CTCCTTAATGTCACGCACGAT |
| Col I* | GAGGGCCAAGACGAAGACATC | CAGATCACGTCATCGCACAAC |
| TGF-β_1_* | GGCCAGATCCTGTCCAAGC | GTGGGTTTCCACCATTAGCAC |
| Sirt1* | TAGCCTTGTCAGATAAGGAAGGA | ACAGCTTCACAGTCAACTTTGT |

*: these genes were homo species.

Supplementary Table 2. Basic characteristics of Akita mice

|  | WT (n=6) | Akita+NS (n=8) | Akita+Nam (n=6) | F value | P value |
| --- | --- | --- | --- | --- | --- |
| BW (g) | 27.67±2.35 | 22.80±2.13^**^ | 21.30±1.56^***^ | 13.70 | <0.001 |
| BG (mM) | 6.13±0.73 | 28.89±4.82^***^ | 26.83±5.49^***^ | 46.51 | <0.001 |
| TCH (mM) | 2.75±0.15 | 4.17±0.17 | 4.81±0.27 | 3.249 | 0.07 |
| TG (mM) | 1.64±0.08 | 2.34±0.10 | 2.69±0.12 | 2.594 | 0.11 |
| KW/BW (mg/g) | 25.99±3.90 | 25.63±2.25 | 25.09±3.31 | 0.10 | 0.91 |
| SBP (mmHg) | 113.83±14.93 | 110.00±4.20 | 97.67±23.49 | 1.95 | 0.17 |
| DBP (mmHg) | 76.33±15.24 | 70.36±15.90 | 69.78±23.75 | 0.23 | 0.80 |

BW: blood weight, BG: blood glucose, TCH: total cholesterol, TG: triglyceride, KW: kidney weight, SBP: systolic blood pressure, DBP: diastolic blood pressure.

^***^ means P<0.001; ^**^ means P<0.01; compared to WT group

Data are mean ± SEM.
