# Supplementary material for: Nicotinamide protects against diabetic kidney disease through regulation of Sirt1
Source: Endocrine. 2024 Mar 6;85(2):638–48. doi: 10.1007/s12020-024-03721-7 (PMC11291543; doi:10.1007/s12020-024-03721-7)
Supplement: Supplementary file 3 — Supplementary figures [file 12020_2024_3721_MOESM3_ESM.docx]

Supplementary Figure 1. Nam reduces urinary albumin and proximal tubular injury in diabetic kidney. (A) The UACR, (B) serum creatinine levels and (C) urinary KIM-1/uCr in Akita diabetic mice. ** p < 0.01; *** p < 0.001; UACR, urinary albumin-to-creatinine ratio; uCr, urinary creatinine; WT, nondiabetic control; Akita+NS, diabetic control, Akita+Nam, diabetic mice treated with Nam.

Supplementary Figure 2. Nam protected the kidney structure in DKD. Representative images of renal tissue stained with (A) HE, (B) PAS and (C) Masson in all mice. (D) Representative electronic micrographs of the GBM and fenestrae of endothelial cells. Red arrow: basement membrane, yellow arrow: fenestrae of endothelial cells, blue arrow: foot process of podocyte. (E) Representative electronic micrographs of the tubules. (F, G) The glomerular sclerosis indexes for PAS and Masson staining, respectively. (H, I) The quantitative thickness of the GBM and foot process width for these 3 groups. (J) Protein expression of Col IV for 3 groups of mice. (K) Gene expression of Col IV for 3 groups of mice;** p < 0.01; *** p < 0.001; WT, nondiabetic control; Akita+NS, diabetic control, Akita+Nam, diabetic mice treated with Nam.

Supplementary Figure 3. Nam has no impact on the body weight and blood glucose of the diabetic mice. (A) The weekly BW records of the mice. (B) The weekly BG records of the mice. * p < 0.01 compared with STZ+Nam group; ** p < 0.001 compared with STZ+Nam group; NDM, nondiabetic control; STZ+NS, diabetic control, STZ+Nam, diabetic mice treated with Nam.

Supplementary Figure 4. Nam has no impact on the renal expression of Sirt3 and Sirt6. (A) Gene expression of Sirt3 for 3 groups of mice; (B) Gene expression of Sirt6 for 3 groups of mice;* p < 0.05; ** p < 0.01; NDM, nondiabetic control; STZ+NS, diabetic control, STZ+Nam, diabetic mice treated with Nam.
